# Refinements to Animal Models for Biomedical Research

**DOI:** 10.3390/ani10122425

**Published:** 2020-12-18

**Authors:** Gabrielle C. Musk

**Affiliations:** Animal Care Services, University of Western Australia, Perth 6107, Australia; gabrielle.musk@uwa.edu.au

This collection includes the manuscripts published in the Special Issue of Animals: Refinements to Animal Models for Biomedical Research. Twelve peer-reviewed papers covering a range of approaches to the concept of refinement have been included in this contemporary resource. The principles of the 3Rs (reduction, refinement and replacement) form the basis of ethically acceptable research using animal models for human and/or animal benefits. If alternatives to the use of animals (replacement) are not available, the importance of reduction and refinement cannot be overstated. 

Reduction and refinement are closely linked. One contributor to the number of animals maintained in a study is the frequency and severity of unexpected or adverse events, which may result in the animal being excluded from the study. Refinement should not only result in optimisation of well-being and welfare of research animals, but should also mitigate the risk of the unplanned occurring, which will in turn directly reduce the number of animals wasted in the process. Society, funding bodies and governments all agree that adverse effects such as pain, fear and distress should be appropriately mitigated and that animals should be housed in conditions that promote their health and well-being [1]. Opportunities for refinement should be continuously sought and explored to meet the expectations of these stakeholders, to benefit the animals involved and to reduce the number of animals used overall.

Nevertheless, what is refinement? The original definition of refinement proposed by Russell and Burch (1959) was “any decrease in the incidence or severity of inhumane procedures applied to those animals which still have to be used” [2]. These authors also stated that the “object of refinement is simply to reduce to an absolute minimum the amount of distress imposed on those animals that are still used” [2]. Despite the apparent inconsistency—is it “any decrease” or is it “to reduce to an absolute minimum”?—and along with various other contemporary definitions, the overriding aim of refinement should be that, whenever possible, the elimination of animal distress consistent with the conduct of sound science is both a scientific and ethical aim [3].

With this aim in mind, it is worth reinforcing that refinements should be evidence-based wherever possible. This collection of manuscripts contributes to the body of evidence to support the introduction of refinements in a range of species including mice [4], rats [5], rabbits [6], guinea pigs [7], pigs [8] and sheep [9]. The systematic review included herein by Whittaker and Barker investigated the question of the impact of blood sampling on the well-being of mice and the quality of the blood sample [4]. Unfortunately, the findings of the review were largely inconclusive as the nature of the primary research introduced a high risk of bias. These authors acknowledge that despite significant and repeated efforts to improve the standard of reporting of the use of animals for scientific purposes, there is still much to be achieved [4]. Adoption of the principles within the Animal Research: Reporting of In Vivo Experiments (ARRIVE) and Planning Research and Experimental Procedures on Animals: Recommendations for Excellence (PREPARE) guidelines, amongst other such recommendations, will help achieve more rigorous, transparent and transferable animal-based research [10,11]. Unfortunately, these principles are still not commonly applied and strong evidence to support proposed methods of animal care, especially for the purpose of refinement, is difficult to collate at the current time. Nevertheless, refinements can still be made and in the process the body of evidence will grow. In the future, the basis for refinements will hopefully shift from less of an empirical mode to more of an evidence-based approach. In turn, there may be stronger justification to standardize aspects of animal care between projects performed at different locations which will improve our capacity to compare results. For example, Kint et al. describe such an approach for the anaesthesia of rats undergoing functional magnetic resonance imaging in this Special Issue [5].

Further consideration of Russell and Burch (1959) and their opinion that refinement may be considered a distinct method of removing inhumanity, focusing on the actual conduct of research and how sentient animals are treated [3]: the indisputable value of anaesthesia and analgesia in refinements in animal experimentation has been acknowledged for some time [12]. Clutton provides a comprehensive Anglocentric history of anaesthetics and analgesics in the refinement of animal experiments in this Special Issue. The importance of anaesthesia as the greatest single advance in humane technique is described along with the role of anaesthetics and analgesics prescribed and administered by appropriately trained people being acknowledged [12]. This comprehensive commentary of Clutton’s is thoroughly researched and is a useful resource to understand the scope of progress that has been made in the use of anaesthetics and analgesics in general, but specifically in the care of research animals.

Other publications in this Special Issue provide useful practical contributions to the often sparse evidence for the use of analgesia for animals, relating to the use of transdermal fentanyl patches in rabbits [6] and sheep [9]. The greatest challenge in providing analgesia for animals is pain assessment and, within this book, the utility of pain assessment strategies is also discussed. The use of nociceptive threshold testing in multiple species is described [13] along with the value of facial grimace scales [14,15]. In addition, the quest for an optimal approach to pain assessment in neonatal piglets, in the context of castration, is described [8]. Perhaps the ultimate aim of pain assessment for both individual animals and for groups of animals in analgesic efficacy studies is an objective measurement. Circulating compounds such as inflammatory cells may be useful and Kongara et al. describe quantification of gene expression of peripheral leucocyte inflammatory cytokines in calves undergoing disbudding [16]. This collection of papers describes the evolution of various approaches to pain assessment in animals with constructive commentary on how to exploit their use. Ongoing efforts to develop strategies for pain assessment in animals are necessary until we are able to reliably and consistently identify and interpret signs or markers of pain in animals.

In classic laboratory animal species, the health status of the animal is well documented and understood. Some species, including sheep (and pigs), are not usually bred specifically for research and are often sourced from commercial farming enterprises. Berset et al. present the results of a European survey, which elucidated the importance of health status documentation and monitoring as essential components to refinement in the use of sheep [17]. These authors highlight the frequency and negative impact of pre-existing animal health issues on the research objectives.

A single manuscript on refinements for euthanasia was also included in this Special Issue. The case study explores the use of an irreversible penetrating spring-loaded captive bolt method of euthanasia in guinea pigs with favourable results [7]. This manuscript is a good example of the translation of techniques from one species to another and the collation of preliminary evidence to establish safety and efficacy in a novel species.

The contents of this Special Issue are evidence for the enthusiasm for the concept of refinement across a broad range of species and contexts within biomedical and animal research.

## References

[1] Prescott M.J., Lidster K. (2017). Improving quality of science through better animal welfare: The NC3Rs strategy. Lab. Anim..

[2] Russell W.M.S., Burch R.L. (1959). The Principles of Humane Experimental Technique.

[3] Tannenbaum J., Bennett B.T. (2015). Russell and Burch’s 3Rs then and now: The need for clarity in definition and purpose. J. Am. Assoc. Lab. Anim. Sci..

[4] Whittaker A.L., Barker T.H. (2020). The Impact of Common Recovery Blood Sampling Methods, in Mice (Mus Musculus), on Well-Being and Sample Quality: A Systematic Review. Animals.

[5] Kint L.T., Seewoo B.J., Hyndman T.H., Clarke M.W., Edwards S.H., Rodger J., Feindel K.W., Musk G.C. (2020). The Pharmacokinetics of Medetomidine Administered Subcutaneously during Isoflurane Anaesthesia in Sprague-Dawley Rats. Animals.

[6] Mirschberger V., von Deimling C., Heider A., Spadavecchia C., Rohrbach H., Zeiter S. (2020). Fentanyl Plasma Concentrations after Application of a Transdermal Patch in Three Different Locations to Refine Postoperative Pain Management in Rabbits. Animals.

[7] Cohen S., Kwok M., Huang J. (2020). Humane Euthanasia of Guinea Pigs (Cavia porcellus) with a Penetrating Spring-Loaded Captive Bolt. Animals.

[8] Sheil M., Polkinghorne A. (2020). Optimal Methods of Documenting Analgesic Efficacy in Neonatal Piglets Undergoing Castration. Animals.

[9] Buchholz T., Hildebrand M., Heider A., Stenger V., Arens D., Spadavecchia C., Zeiter S. (2020). Transdermal Fentanyl Uptake at Two Different Patch Locations in Swiss White Alpine Sheep. Animals.

[10] Percie du Sert N., Hurst V., Ahluwalia A., Alam S., Avey M.T., Baker M., Browne W.J., Clark A., Cuthill I.C., Dirnagl U. (2019). The ARRIVE guidelines 2019: Updated guidelines for reporting animal research. bioRxiv.

[11] Smith A.J., Clutton R.E., Lilley E., Hansen K.E.A., Brattelid T. (2018). PREPARE: Guidelines for planning animal research and testing. Lab. Anim..

[12] Clutton R.E. (2020). An Anglocentric History of Anaesthetics and Analgesics in the Refinement of Animal Experiments. Animals.

[13] Taylor P. (2020). Remote Controlled Nociceptive Threshold Testing Systems in Large Animals. Animals.

[14] Cohen S., Beths T. (2020). Grimace Scores: Tools to Support the Identification of Pain in Mammals Used in Research. Animals.

[15] Mota-Rojas D., Olmos-Hernández A., Verduzco-Mendoza A., Hernández E., Martínez-Burnes J., Whittaker A.L. (2020). The Utility of Grimace Scales for Practical Pain Assessment in Laboratory Animals. Animals.

[16] Kongara K., Dukkipati V.S.R., Tai H.M., Heiser A., Murray A., Webster J., Johnson C.B. (2020). Differential Transcription of Selected Cytokine and Neuroactive Ligand-receptor Genes in Peripheral Leukocytes from Calves in Response to Cautery Disbudding. Animals.

[17] Berset C.M., Lanker U., Zeiter S. (2020). Survey on Sheep Usage in Biomedical Research. Animals.

